# The Legitimacy for the Use of Splint Immobilization After Elastic Intramedullary Nailing of Both-bone Forearm Shaft Fractures in Children

**DOI:** 10.5435/JAAOSGlobal-D-25-00102

**Published:** 2025-10-16

**Authors:** Stanisław Kłosiński, Marek Synder, Robin Novriansyah, Danendra Rakha Putra Respati, Andrzej Borowski

**Affiliations:** From the Clinic of Orthopaedics and Paediatric Orthopaedics, Medical University of Lodz, Lodz, Poland (Mr. Kłosiński, Mr. Synder, and Mr. Borowski), and the Departement of Surgery, Faculty of Medicine, Universitas Diponegoro, Semarang, Indonesia (Mr. Novriansyah and Mr. Respati).

## Abstract

**Background:**

Pediatric diaphyseal both-bone forearm fractures are increasingly treated with elastic stable intramedullary nailing (ESIN) using titanium nails. However, clear guidelines on the type and duration of splint immobilization are lacking. This study aims to assess the necessity of splint immobilization after ESIN for diaphyseal forearm fractures in children.

**Methods:**

A prospective study was conducted on 38 patients with isolated radial and ulnar shaft fractures from 2018 to 2020. Patients were divided into two groups: 14 with splint immobilization for a mean of 3.93 weeks and 24 with only a sling allowing early postoperative movement. The mean ages were 10.3 years (group I) and 10.0 years (group II). Patients were evaluated at 2, 6, 12, and 24 weeks postsurgery. Final range of motion, recovery pace, bone healing, pain, complications, and treatment outcomes were compared.

**Results:**

No notable differences were found between splint immobilization and nonsplint groups regarding bone healing time (3.79 vs. 3.13 months), complications (28.6% vs. 29.2%), and final outcomes. Movement recovery was faster in the nonsplint group, but range of motion was similar at the final follow-up. Pain-free rates at 2 weeks postsurgery were higher in the splint group (85.8% vs. 50%). Pain intensity was similarly low in both groups (mean 2.5 vs. 2.67 on the visual analog scale scale).

**Conclusion:**

Splint immobilization improves pain control in the first 2 weeks postsurgery, but it offers no additional benefits in terms of healing time and functional outcome. Meanwhile, ESIN is a safe, effective treatment for pediatric forearm shaft fractures.

Fractures of the forearm are among the most common injuries in pediatric population and account for about 40% of all fractures that occur in children. Most occur at the distal third but middle and proximal thirds also.^1^ For restoring anatomical alignment and limb function, preventing complications and promoting speedy healing, maintaining these are the major aims when treating forearm fractures in children.^2^

The standard procedure for most forearm shaft fractures is closed reduction followed by immobilization with plaster splint.^3^ Normally, conservative treatment yields good results because of the excellent healing potential and children's bones capability to remodel themselves over time.^4^ Nonetheless, there are also risks associated with it. In particular, such dangers include fracture redisplacement, loss of reduction, and malunion which may occur especially in the event of an unstable or severely displaced fracture.^5^

Some circumstances may need surgical operation as the best option to get desirable results. The reasons for surgery are compound, unstable, or displaced fractures or when closed reduction was not successful.^2^ Elastic stable intramedullary nailing (ESIN) has become a popular method for surgical treatment of radius and ulna fractures in children.^6^ ESIN provides strong internal fixation where early motion of the limb is possible and it has shown high healing rate of the fracture with good functional outcomes. However, ESIN also has disadvantages, such as risk of infection, implant problems, and secondary procedure for removal of the nails.^7,8^

The course of action after ESIN is postoperative care that includes immobilization of the limb for proper healing and preventing complications. Although a splint is usually used, questions have arisen concerning its necessity in recent times. Some doctors argue for splint immobilization as a matter of routine, asserting that it avails pain relief and shields the surgical spot from injury while guarding against refracture among others.^9,10^ On the other hand, some people are convinced that there may be no need of having their wide use across all cases with early mobilization without splint leading to improved functional and quick return to normal activities.^11^

This study was triggered by the controversy surrounding the need to provide plaster splint immobilization after ESIN in children. Hence, this study aims at comparing these two groups to ascertain if regular use of splint immobilization is justified or can be safely omitted without negatively affecting the healing process and functional outcomes.

## Methods

### Study Design

Data gathering for this project has been conducted in a prospective manner over 2018 to 2020 among patients who received ESIN according to the Métaizeau method because of diaphyseal forearm fractures. Their electronic medical records, which included imaging results, doctor's conclusions, other studies, and outpatient clinic archives, were used. All underaged patients' legal representatives signed their written consent forms before they could take part in the research. Before beginning the study, approval by Bioethics Committee was received as well as from the hospital authorities.

Patients were divided into two homogeneous groups: splint immobilization and without splint immobilization (sling with early movement allowed). The doctor performing the surgery determined the type of immobilization and the physician in outpatient clinic chose its duration. After being assigned to each group, patients were monitored at the interval of 2, 6, 12, and 24 weeks in the outpatient clinic.

### Patients Criteria

The inclusion criteria consisted of age between 3 and 16 years, body weight less than 50 kg, diagnosis of a middle third radius and ulna diaphyseal fracture, and indication for surgical treatment with ESIN. Furthermore, there should have been no previous fractures or surgeries on the same limb, and consents must have been signed by patients' parents. Patients were excluded from the study if they had pathological fractures because of bone tumors or metabolic bone diseases, open fracture, fractures involving parts other than the forearm, fractures fixation of only one forearm bone, and fracture with dislocation of the humeroradial or distal radioulnar joints. In addition, any of the following conditions** **were considered exclusion criteria: nerve/vessels injuries such as those which could alter outcomes of this research; lack of compliance to postoperative protocol; and scheduled appointments after surgery.

### Surgery and Postoperative Care

Every patient underwent surgery under general anesthesia by a group of experienced orthopaedic surgeons using the standard ESIN technique. First, closed reduction of fracture was performed under fluoroscopy. In case it failed, a minor open reduction would be done. After an acceptable reduction, a small incision was made at the proximal or distal metaphysis of the radius and/or ulna depending on where the fracture is located. It is only now that flexible intramedullary nails were inserted into the medullary canal and advanced across the fracture site under fluoroscopic guidance to ensure proper alignment and stability. Nails were carefully advanced up to the metaphyseal level on opposite bone end. These nails were then cut so that there was just a small exposed part for easy removal. Finally, some sutures were used to close up these incisions as well as sterile dressings.

At the end of surgery, the immobilization group received a long arm splint, typically worn for 4 to 6 weeks depending on progress and the surgeon's judgment. Acetaminophen was given at the same dose of 15 mg/kg in all patients, 3 times a day, only for the first 3 days after surgery. Once the splint was removed, patients were given instructions on exercises to restore full range of motion (ROM), by the doctor in outpatient clinic (flexion-extension of elbow and supination-pronation of forearm). The nonimmobilization group did not receive a splint but was provided with a sling. These patients began gentle mobilization exercises (the same as abovementioned) on the first day after surgery under physiotherapist supervision and were discharged from hospital with advice on how to protect their limbs during early recovery and continue exercises at home few times a day.

### Outcomes Measures

The main outcomes measured in the study comprised several essential features of recovery, including: (1) rate of recovery of full ROM of the elbow and forearm joint; (2) time required for bone union to occur; (3) degree of pain associated with treatment; (4) frequency and type of postoperative complication; and (5) final outcome.

Recovery of ROM was assessed by the author using a goniometer during follow-ups, with 85° for pronation/supination and 140-0° for elbow flexion-extension considered normal.^12-14^ Treatment success was categorized based on rotational motion loss: very good (no loss), good (<10° loss), average (10° to 30° loss), and poor (>30° loss).^15-18^ Radiological evaluations at 6 and 12 weeks determined bone healing, defined by bridging callus (6 weeks) and adequate trabecular formation (12 weeks). Healing beyond this period was delayed union, whereas nonhealing after 6 months was nonunion.^8,19,20^ Pain levels were assessed using the visual analog scale (VAS; 0 to 10), incorporating pictograms for younger patients. Complications such as infections, implant issues, and neurovascular problems were classified using a modified Clavien-Dindo system; severe complications were grades III–V proposed by Martus et al.^20^

The final treatment outcome was categorized into four groups: very good, good, average, and poor, based on clinical, radiological, and functional criteria. This system was developed by Martus et al,^20^ but includes several modifications for a more precise reflection of the results.^2,21-23^ Very good outcomes indicate full ROM with no or minor (grade 1) complications, while good outcomes involve less than 10° ROM loss in pronation/supination and complications up to grade 2. Average outcomes correspond to 10° to 30° ROM loss with complications up to grade 3, whereas poor outcomes are characterized by more than 30° ROM loss and severe complications (grade 4 to 5). This classification provides a structured and objective evaluation of treatment success.

### Statistical Analysis

Statistical analysis was conducted using Statistica software. Continuous variables were presented as mean ± standard deviation (SD), whereas categorical variables were shown as percentages. The chi square test was used for categorical data, and the Student *t*-test or Mann-Whitney *U* test was applied for continuous data, depending on normality, which was assessed using the Shapiro-Wilk test. Owing to notable deviations from normality, nonparametric tests were used.

The Mann-Whitney *U* test compared means between immobilized and nonimmobilized patients. Mobility recovery at weeks 2, 6, 12, and 24 was analyzed using Friedman analysis of variance, with the Wilcoxon signed-rank test for post hoc analysis. Box and whisker plots displayed means, SDs, and range. The chi square test examined qualitative variables, applying Yates correction for small samples and Fisher exact test for very small frequencies. Statistical significance was set at *P* < 0.05.

## Results

### Patients Selection and Characteristic

This study included 38 patients with isolated both-bone forearm shaft fractures, divided into two groups. The first group (n = 14) received splint immobilization on the upper arm, hand, or forearm, whereas the second group (n = 24) had no plaster immobilization and were given a sling with instructions for light rehabilitation.

Both groups were predominantly male, with no significant differences in sex distribution, age, fracture type (open/closed), or fracture side (*P* > 0.05, Table 1). However, men were more common in the nonimmobilization group (66.7%) than in the immobilization group (57.1%). The immobilization group had patients aged 6 to 14 years (mean 10.3 ± 2.76 years), with half aged 10.5 years or older. The nonimmobilization group had patients aged 6 to 16 years (mean 10.0 ± 2.7 years), with half aged 9.5 years or younger.

**Table 1:** Baseline Patients Characteristic

Parameter	With Immobilization (N = 14)	Without Immobilization (N = 24)	*P* Value
Age (yrs)			*P* = 0.770
Min-max	6-14	6-16	
Median	10.5	9.5	
x̄ ± SD	10.5 ± 2.76	10.0 ± 2.70	
Sex (N [%])			*P* = 0.557
Male	8 [57.1]	16 [66.7]	
Female	6 [42.9]	8 [33.3]	
Side of fracture (N [%])			*P* = 0.859
Right	6 [42.9]	11 [45.8]	
Left	8 [57.1]	13 [54.2]	
Duration of immobilization (wks)			N/A
Min-max	2-10	N/A	
Median	3.0	N/A	
x̄ ± SD	3.93 ± 2.76	N/A	
Type of fracture (N [%])			*P* = 0.607
Open	1 [7.1]	1 [4.2]	
Closed	13 [92.9]	23 [95.8]	

### Recovery and Rate of Achieving Full Range of Motion

Elbow flexion in the immobilized group showed significant improvement over time (*P* < 0.001), progressing from initial stiffness to full extension by week 24. Significant differences were noted between weeks 2 and 6, 2 and 12, and 2 and 24 (all *P* < 0.01), as well as between weeks 6 and 12 and 6 and 24 (both *P* < 0.05), but not between weeks 12 and 24 (*P* > 0.05). Similarly, in the nonimmobilized group, elbow extension improved significantly (*P* < 0.001) from hyperextension to negative values, with major changes between weeks 2 and 6, 2 and 12, and 2 and 24 (all *P* < 0.001). Changes also occurred between weeks 6 and 12 and 6 and 24, whereas no significant differences were observed between weeks 12 and 24 (Table 2).

**Table 2:** Comparison of the Recovery of Full Range of Motion at Different Weeks Between Immobilized and Nonimmobilized Patients

Factor or Variable	Week	With Immobilization (N = 14)			Without Immobilization (N = 24)			Test Value (z)	*P* Value
		Median	x̄ ± SD	V%	Median	x̄ ± SD	V%		
Elbow flexion	2	120.0	115.7 ± 8.96	7.7	120.0	124.4 ± 11.5	9.2	1.816	0.069
	6	140.0	138.7 ± 12.4	8.9	140.0	140.0 ± 5.62	4.0	0.182	0.856
	12	145.0	143.6 ± 8.42	5.9	140.0	143.1 ± 4.62	3.2	0.711	0.477
	24	145.0	145.0 ± 5.19	3.6	142.5	144.0 ± 4.42	3.1	0.514	0.607
Elbow extention	2	−22.5	−27.1 ± 17.4	64.2	−20.0	−18.8 ± −20.0	72.9	1.165	0.244
	6	0.0	−10.0 ± 17.8	178.0	0.0	−3.04 ± 0.0	223.7	0.862	0.388
	12	0.0	−2.5 ± 7.0	280.0	0.0	0.0 ± 0.0	N/A	0.696	0.486
	24	0.0	0.0 ± 0.0	N/A	0.0	0.42 ± 0.0	485.7	0.197	0.844
Forearm pronation	2	42.5	40.0 ± 17.3	43.3	62.5	56.9 ± 19.8	34.8	2.567	0.0115
	6	65.0	61.6 ± 17.2	28.0	80.0	74.4 ± 17.3	23.3	2.360	0.0183
	12	77.5	77.1 ± 12.2	15.9	90.0	82.3 ± 11.6	14.1	1.695	0.090
	24	90.0	86.6 ± 5.39	6.2	90.0	85.7 ± 8.06	9.4	0.045	0.964
Forearm supination	2	37.5	40.4 ± 20.5	50.8	55.0	52.3 ± 16.7	30.0	1.664	0.096
	6	74.0	65.5 ± 23.8	36.4	80.0	75.8 ± 13.4	17.7	1.498	0.134
	12	85.0	83.2 ± 6.38	7.7	90.0	86.3 ± 5.37	6.2	1.528	0.126
	24	90.0	6.38 ± 2.13	2.4	90.0	89.2 ± 1.90	2.1	0.227	0.820

Elbow flexion in both groups improved significantly (*P* < 0.001). In the immobilized group, notable differences were observed between weeks 2 and 6, 2 and 12, and 2 and 24 (all *P* < 0.01). In the nonimmobilized group, significant progress was also seen (*P* < 0.0001) between weeks 2 and 6 and 2 and 12 (*P* < 0.0001), but not between weeks 6 and 12 or 6 and 24.

Forearm pronation improved significantly in both groups (*P* < 0.001). In the immobilized group, greater improvements were seen between weeks 2 and 6, 2 and 12, and 2 and 24 (all *P* < 0.01), as well as between weeks 6 and 12, 6 and 24, and 12 and 24 (all *P* < 0.01). The nonimmobilized group showed similar improvements (*P* < 0.001), with significant differences in the same time points (all *P* < 0.001).

Forearm supination also showed significant improvement (*P* < 0.001) in both groups. The immobilized group had notable changes between weeks 2 and 6, 2 and 12, and 2 and 24 (all *P* < 0.01), with continued improvement between weeks 6 and 12, 6 and 24 (both *P* < 0.01), and between weeks 12 and 24 (*P* < 0.05). Similar progress was seen in the nonimmobilized group, with significant changes across the same time points (all *P* < 0.001, except *P* < 0.05 for weeks 12 to 24).

Overall ROM recovery was assessed as 75° to 85° forearm rotation and full elbow extension/flexion. No significant differences were observed between groups at weeks 2, 6, and 12 (*P* > 0.05). At week 2, no patients achieved full ROM. By week 6, 7.1% of the immobilized group and 16.7% of the nonimmobilized group attained full ROM. By week 12, the percentages rose to 28.6% and 45.8%, respectively. By week 24, a significant difference emerged (*P* < 0.05), with 64.3% of the immobilized group and 29.2% of the nonimmobilized group reaching full ROM. Some nonimmobilized patients achieved full ROM later or not at all, but this difference was not statistically significant. In conclusion, the nonimmobilized group recovered ROM faster than the immobilized group. The results are summarized in Table 3.

**Table 3:** Comparison of the Rate of Achieving Full Range of Motion at Different Weeks Between Immobilized and Nonimmobilized Patients

Week	Full ROM Achieved in Group (N [%])		Chi Square	*P* Value
	Immobilized (N = 14)	Nonimmobilized (N = 24)		
2	0 [0.0%]	0 [0.0%]	N/A	N/A
6	1 [7.1%]	4 [16.7%]	0.116	0.116
12	4 [28.6%]	11 [45.8%]	0.499	0.499
24	9 [64.3%]	7 [29.2%]	4474	4.474
Later or no full ROM	0 [0.0%]	2 [8.3%]	NA	NA

NA = not applicable; ROM = range of motion

### Radiographic Analysis of Bone Union

Proper fracture healing was defined as primary bridging callus by week 6 and complete union by month 3 after surgery. The average time for complete bone union was faster in the nonimmobilization group (3.13 ± 0.61 vs. 3.79 ± 2.22 months), although the difference was not statistically significant (*P* > 0.05, Table 4).

**Table 4:** Comparison of Required Time for Bone Union, Pain Symptomps, Complication Severity, and Final Outcomes Between Immobilized and Nonimmobilized Patients

Parameter	Immobilized (N = 14)	Nonimmobilized (N = 24)	Comparison
Bone union time			
Average time required (x̄ ± SD)	3,79 ± 2,22	3,13 ± 0,61	z = 0.514; *P* = 0.607
On time 3 months (N [%])	12 [85.8%]	23 [95.8%]	chi^2^ = 1961; *P* = 0.375
Delayed 3-6 months (N [%])	1 [7.1%]	1 [4.2%]	
None >6 months (N [%])	1 [7.1%]	—	
Pain symptoms (2nd week)			
Average VAS (x̄ ± SD)	2.5 ± 0.71	2.67 ± 1.30	z = 0.173; *P* = 0.866
None (N [%])	12 [85.8%]	12 [50%]	*P* = 0.027
Complication severity			
Degree I (N [%])	2 [14.3%]	1 [4.2%]	*P* = 0.302
Degree II (N [%])	1 [7.1%]	4 [16.7%]	*P* = 0.381
Degree III (N [%])	2 [14.3%]	1 [4.2%]	*P* = 0.302
Degree IV (N [%])	1 [7.1%]	1 [4.2%]	*P* = 0.607
Degree V (N [%])	0 [0.0%]	0 [0.0%]	NA
Final outcomes			
Very good (N [%])	9 [64.3%]	14 [58.3%]	chi^2^ = 0.489; *P* = 0.921
Good (N [%])	3 [21.5%]	6 [25.0%]	
Average (N [%])	1 [7.1%]	3 [12.5%]	
Poor (N [%])	1 [7.1%]	1 [4.2%]	
Final loss of pronation and/or supination			
0° (N [%])	11 [78.6%]	21 [87.5%]	chi^2^ = 3.753; *P* = 0.153
<10° (N [%])	3 [21.4%]	1 [4.2%]	
10°-30° (N [%])	0 [0.0%]	2 [8.3%]	
>30° (N [%])	0 [0.0%]	0 [0.0%]	

NA = not applicable, VAS = visual analog scale

The distribution of bone union time also showed no significant difference (*P* > 0.05). In both groups, bone union most commonly occurred by the 3rd month (85.8% [n = 12] in the immobilization group vs. 95.8% [n = 23] in the nonimmobilization group). Delayed union was observed in one patient per group (7.2% vs. 4.2%), while one immobilized patient (7.1%) experienced nonunion, which healed spontaneously in the 11th month.

### Assessment of Pain Symptoms

Pain symptoms were observed only in the 2nd week and were absent in the 6th, 12th, and 24th weeks, so only 2nd-week results were analyzed. Two weeks postoperatively, significant differences in pain levels were found between the groups (χ^2^ = 4.847; *P* = 0.0277). In the immobilization group, 85.8% (n = 12/14) reported no pain (VAS 0), compared with 50.0% (n = 12/24) in the nonimmobilization group.

In the immobilization group, 7.1% (n = 1) reported mild pain (VAS 2) and 7.1% (n = 1) reported moderate pain (VAS 3). In the nonimmobilization group, 8.3% (n = 2) reported very mild pain (VAS 1), 16.7% (n = 4) mild pain (VAS 2 to 3), and 8.3% (n = 2) moderate pain (VAS 5). No patients in either group had severe pain (VAS ≥ 6). Among those with pain, the mean VAS score was 2.5 ± 0.71 in the immobilization group and 2.67 ± 1.30 in the nonimmobilization group. The statistical comparison (*P* = 0.866) confirmed no significant difference in pain intensity between groups.

### Complications

In total, complications occurred in 11 patients (28.9%) across both groups, with similar rates in the immobilization (28.6%, n = 4) and nonimmobilization (29.2%, n = 7) groups (*P* > 0.05). The most common complications were asymptomatic delayed union (7.9%, n = 3) and bursitis from nail irritation (7.9%, n = 3).

No statistically significant differences were found in the frequency of specific complications (*P* > 0.05). Most complications occurred once per group, except for delayed union, which was observed twice in the immobilization group and once in the nonimmobilization group, while bursitis affected three nonimmobilized patients and none in the immobilization group. One immobilized patient experienced three complications: delayed union, flexor tendon entrapment (III and IV fingers), and extensor pollicis longus rupture, leading to prolonged immobilization. Grouping complications by severity showed no significant differences (*P* > 0.05). However, degree I and III complications were more frequent in the immobilization group (14.3% vs. 4.2%), whereas degree II complications were more common in the nonimmobilization group (16.7% vs. 7.1%). No degree V complications were observed.

### Final Outcomes

No statistically significant difference in final mobility loss was found between the groups (*P* > 0.05). The nonimmobilization group more frequently had a 0° loss (87.5% vs. 78.6%) and losses in the 10° to 30° range (8.3% vs. 0.0%), whereas the immobilization group more often experienced minor losses up to 10° (21.4% vs. 4.2%). No patients in either group lost more than 30°.

Correlating final mobility loss with complications, no significant difference in treatment outcomes was found (*P* > 0.05). Very good results were most common, slightly higher in the immobilization group (64.3% vs. 58.3%). Good outcomes were similar (21.5% vs. 25.0%), and average results were less frequent in the immobilization group (7.1% vs. 12.5%). Poor outcomes were rare, with one patient in each group (7.1% vs. 4.2%). One patient had a successful tendon transplant, while another awaited surgery. In summary, good or very good recovery (final loss of 0° or <10°) was observed in most patients (94.7%), although this dropped to 84.2% when considering treatment complications.

## Discussion

In the literature, it is challenging to find studies based on a scheme similar to this study, comparing treatment outcomes depending on the type of immobilization used. Most studies only discuss various concepts regarding the time and type of plaster immobilization used after fixation with the ESIN technique ranging from no immobilization at all, to a splint, or full splint (Table 5). In addition, a significant majority of the referenced studies did not address the pain associated with the method and the potential immobilization. Considering the above statements, it seemed justified to conduct a study that compares the advantages and disadvantages of possible variants.

**Table 5:** Summary of Previous Studies Outcomes on Postoperative Immobilization After Elastic Stable Intramedullary Nailing

Author	Design	N	Age; Mean (Range)	Population	Intervention	Type of Immobilization	Outcomes
Lascombe et al^24^	Cohort	76	Children, age is not specified	Forearm shaft fractures. Type of fracture is not specified	ESIN followed by immediate immobilization for 5-6 weeks, then sling for 2-3 week	Plaster cast (5-6 weeks), followed by sling for 2-3 weeks	In a 3.5-year follow-up of 76 patients, 92% achieved excellent outcomes, including complete restoration of range of motion
Verstreken et al^25^	Case series	6	10 (5-13) yrs	Complete displaced forearm shaft fracture who failed conservative treatment	ESIN followed by immediate mobilization. All patients were discharged home within 1 or 2 days after surgery. Sports activities were restricted for 2 months	No cast/splint; early mobilization	No delays in bone healing or repeat fractures were observed. All patients maintained a full ROM after surgery at 1 year follow-up
Peterlein et al^26^	Cohort	127	8.3 (1-18) yrs	Diaphyseal forearm fractures	ESIN followed by immediate immobilization. Duration of immobilization was not specified	Bandage or sling only	At 12.4-year follow-up, most patients showed good long-term function, with low DASH scores and high Mayo Wrist Scores
Pogorelica et al^27^	Cohort	173	11 (3-17) yrs	Closed and open forearm fracture, including pathological, dislocated, and monteggia fracture	ESIN followed by immediate mobilization, physical therapy, and cryotherapy	No cast/splint; early mobilization	Complications were encountered in 15 patients (8.7%), of whom only 3 required readmission (2 recurrent fractures and 1 pseudarthrosis). All patients achieved full ROM at 6 months follow-up
Kapila et al^28^	Cohort	50	6-14 yrs	Both-bone forearm diaphyseal fractures	ESIN followed by early range of motion exercises. Splint was used depending on the stability of the fixation and number of fragments, some patients received plaster splint for up to 3 weeks	Type of cast or splint (forearm/full arm) is not specified	Radiological bone union was achieved by 9.2 weeks. At the 6-month follow-up, four patients experienced mild reductions in forearm supination and pronation, with no major complications reported
Fernandez et al^8^	Cohort	553	9.1 (4-16) yrs	Forearm fractures of the middle third or the transition zones	ESIN with/without immobilization. Full fitted forearm plaster cast for 2-4 weeks postsurgery only given for patients with fractures in the distal third of the forearm shafts (n = 116)	Full arm cast	Complications included 5 wound infections, 1 osteomyelitis, 7 ulnar nonunions, 14 delayed unions, 6 loss of correction, 15 radial nerve injuries, 1 nail malplacement, 5 skin perforations, and 27 refractures
Salonen et al^29^	Cohort	35	12.3 (5.2-17.4) years	Simple both-bone forearm fracture; simple radius fracture; and simple wedge fracture	ESIN followed by immediate immobilization up to 5 weeks. Sports activities were restricted to 2 months	Full arm cast (n = 14), forearm cast (n = 11), and sling (n = 10)	Twenty-three (66%) patients had full union without ROM limitation. Complications occurred in 34% of cases—20% were transient, while 14% were long-term
Kruppa et al^30^	Cohort	201	9.7 (3–16) yrs	Diaphyseal both-bone forearm fractures	ESIN followed by immediate immobilization. The duration is depending on the intraoperative fracture stability, individual surgeon's decision, and wound healing process	Full arm cast (until the wound healed), followed by a short cast (2-4 weeks)	The complication rate was 8.9%, with 6.9% requiring readmission and revision surgery, mainly because of refractures, malunions, tendon ruptures, and limited motion
Garg et al^6^	Cohort	21	12.8 (12-21.5) mo	Displaced and unstable diaphyseal forearm fractures	ESIN followed by immediate immobilization for 2-4 weeks	Full arm cast	Union was achieved within 13 weeks in 19 patients. One patient had delayed union and another required bone marrow injection. All patients achieved full ROM eventually
Kapoor et al^31^	Cohort	47	5-15 yrs	Displaced diaphyseal forearm fractures	ESIN followed by immediate immobilization for 6 weeks	Full arm cast	Full ROM was achieved in 80.8% of the patients, 19.1% patients had ROM restriction up to 20° (pronation), considered as good results. Complications occurred in 21.2%, with 14.9% requiring revision surgery
Lyman et al^32^	Cohort	86	<16 yrs	Diaphyseal forearm fractures	ESIN followed by immediate immobilization. The duration is depending on the surgeon's decision, range is not specified	Full arm cast or forearm cast	Twenty-four percent of patients experienced complications, with 12.8% requiring readmission or surgical intervention. Ninety-four percent of patients returned to full limb function at the last follow-up visit
Colaris et al^33^	Cohort	24	<16 yrs	Displaced and unstable both bone diaphyseal forearm fracture	Single-bone (n = 11) vs. both-bone (n = 13) fixation in both-bone fracture using ESIN. Followed by immediate immobilization for 3 weeks in both bone fixation group and 6 weeks in single bone fixation group	Full arm cast	Redisplacement of the fracture occurred in four cases of single-bone nailing group. At the 9-month follow-up, both groups showed a median pronation/supination limitation ranging from 5° to 10°.

DASH = disabilities of arm, shoulder, and hand; ESIN = elastic stable intramedullary nailing; ROM = range of motion

Lascombe et al^24^ initially immobilized 29 of 76 patients for 5 to 6 weeks, later switching to a 2 to 3 week sling, likely because of no observed benefit of rigid immobilization. For the subsequent patients, this was replaced by a sling worn for 2 to 3 weeks. Their 92% very good outcomes were slightly better than those in this study, with 16% complication rates. However, the reason for this change and the specific type of immobilization (splint/cast) were not specified. It can be inferred that the authors saw no benefit of rigid immobilization over a sling and, encouraged by the outcomes, discontinued plaster use for subsequent patients.

Several studies also support early mobilization post-ESIN, suggesting that rigid immobilization may not be necessary. Verstreken et al^25^ and Pogorelica et al^27^ reported excellent outcomes, including full ROM and low complication rates despite not using postoperative casts. Similarly, Peterlein et al^26^ used only slings or bandages, observing favorable long-term functional results (mean follow-up of 12.4 years). Assessment was done using Disabilities of Arm, Shoulder, and Hand and Mayo Wrist Score questionnaires, with the highest scores achieved in 77% and 82% of cases.^26^

Other studies employed routine full arm casting on displaced or unstable forearm fracture. Kruppa et al,^30^ Kapoor et al,^31^ Garg et al,^6^ and Colaris et al^33^ all used long-arm casts on displaced or unstable forearm fracture for 2 to 6 weeks, but this did not consistently correlate with fewer complications. In fact, Garg reported a 57% complication rate, although most were transient.^6^ Kapoor's results were more favorable, with 80.8% achieving full ROM and 21.2% experiencing complications.^31^ As observed, the applied immobilization was not associated with a lower incidence of complications.

The choice of immobilization may also depend on those with residual instability after nailing such as when the nail used is smaller than recommended or when the fracture is located in the proximal-third or distal-third of the shaft immobilization with a cast may be necessary. Kapila et al^28^ used splints selectively based on fracture complexity and fixation stability, noting that even limited immobilization (up to 3 weeks) yielded high satisfaction and recovery rates. Meanwhile, Fernandez et al^34^ reserved short-term plaster casting only for distal third fractures, citing higher instability in that region. In another 2005 study comparing ESIN vs. plate fixation, Fernandez negated the necessity of using plaster after intramedullary nailing of forearm shaft fractures.^34^

Salonen et al^29^ and Lyman et al^32^ used various immobilization types in each different patients depending on the surgeon choices. Salonen's study noted a 34% complication rate; this was the only cited study reporting a flexion contracture of the third finger on the fracture side, attributed to Volkmann's ischemic contracture, requiring surgery in a hand surgery department, with a good final outcome. By contrast, this study linked the condition to tendon entrapment in the bone scar, which resolved with rehabilitation and did not require revision surgery.^29^

Sinikumpu and Serlo^35^ briefly addresses the topic of cast immobilization in his work analyzing current approaches to treating forearm fractures in children. He only mentions that it should be removed for radiological assessment of the fracture at 4 to 6 weeks after surgery and notes that some authors do not recommend plaster immobilization after ESIN. He cites a postoperative complication rate of up to 30%, including transient short-term complications. This result is very close to this study's observations (28.9%).^35^

The incidence of complications varies widely across studies, likely due to differences in assessment criteria and experience levels at different centers. A clear trend shows improved outcomes and fewer complications in hospitals with long-term experience. Larger studies emphasize that, although ESIN may seem straightforward, many complications can be avoided by strictly adhering to surgical techniques.^8,29,36-38^ In the author's observations, a relatively high complication rate (29%) was noted, likely because of the clinic's learning curve, with multiple operators performing around 25 procedures annually over the past 5 years. Similarly, Lyman reported a 24% complication rate with 3 to 14 procedures per year over 9 years.^31^ Garg observed even more complications, with 57% in a small group of 21 operated patients.^6^

Besides conventional plaster cast and splint, another option for postoperative immobilization is to use Velcro splint and personalized three-dimensional (3D)–printed arm cast. Velcro is a removable rigid splint with a hook-and-loop fastener, typically made of lightweight material that can be used alone or along with a plaster cast.^39^ Xu et al^40^ compared plaster cast with the use of a 3D-printed arm cast combined with velcro splint in pediatric distal radius fractures. Patients expressed greater satisfaction with the 3D-printed cast, as reflected in the comfort-related items of the Patient Satisfaction Questionnaire. However, no significant differences were observed in palmar tilt (*P* = 0.196), ulnar deviation (*P* = 0.460), or radial styloid height (*P* = 0.111).^40^

This study, along with findings from other authors, suggests that routine cast immobilization after ESIN is beneficial only to reduce pain in the first 2 weeks after surgery. However, some limitations must be acknowledged. The small sample size makes it difficult to generalize findings, necessitating further research with a larger cohort for validation. In addition, nonrandomized participant assignment may introduce bias, and the 6-month follow-up period may not fully capture long-term treatment outcomes, requiring extended observation over 1 or 2 years for a better assessment of durability. Finally, this study did not include detailed data regarding fracture configuration, reduction quality, nail-to-shaft diameter ratio, or precise fracture location, limiting the ability to analyze these potentially important variables.

## Conclusion

Plaster immobilization after ESIN fixation does not provide any benefits except that it may give a better pain control during 2 first weeks after surgery, but pain resolves regardless in both groups. Although it may initially slow motion recovery, both groups regain comparable mobility by 24 weeks. The ESIN method is safe and effective, with minimal complications. Routine plaster use is unnecessary and should be limited to managing early postoperative pain. Patients can achieve full recovery through regular activities without professional therapy, making immobilization a case-by-case decision.
